# The efficacy and safety of a proposed herbal moisturising cream for dry skin and itch relief: a randomised, double-blind, placebo-controlled trial- study protocol

**DOI:** 10.1186/1472-6882-13-330

**Published:** 2013-11-25

**Authors:** Dong-Hyo Lee, Eun-Sung Seo, Jin-Tae Hong, Gang-Tai Lee, Young-Kyoung You, Kun-Kook Lee, Ga-Won Jo, Nam-Kwen Kim

**Affiliations:** 1 Opthalmology Otolaryngology & Dermatology, Woosuk University Korean Medicine Hospital, Jeonju, South Korea; 2 Graduate School Department of Food and Nutrition, Seoul National University, Seoul, South Korea; 3 College of Pharmacy and Medical Research Centre, Chungbuk National University, Chungbuk, South Korea; 4 Coreana Songpa R&D Centre, Cheonansi, Chungnam, South Korea; 5 Opthalmology Otolaryngology & Dermatology, Wonkwang University Sanbon Oriental Medical Centre, Gunpo, South Korea

**Keywords:** Herbal moisturising cream, Dry skin, Itch relief, Randomised controlled trial

## Abstract

**Background:**

Moisturisers prevent and treat dry skin. They can also protect sensitive skin, improve skin tone and texture, and mask imperfections. Herbal medicines or their extracts have been available as topical formulations and cosmetics. *Arctium lappa L. (Asteraceae)* has been used to treat inflammatory disorders and various skin problems. It could be a candidate herbal medicine for treating dry skin condition.

This study aims to establish the efficacy and safety of a proposed herbal moisturising cream containing *Arctium lappa L.* seed extract, which has been approved by the Korean Ministry of Food and Drug Safety for use in cosmetics.

**Methods/Designs:**

This study is a randomised, double-blind, placebo-controlled study with two parallel groups (proposed herbal moisturising cream vs. placebo cream). We will recruit 66 healthy male and female participants, aged 20 to 65 years, who have been diagnosed with dry skin conditions. Participants will be randomly allocated to receive either the proposed herbal moisturising cream or a placebo cream for four weeks. Each participant will be examined for signs and symptoms before and after using the cream. Skin hydration, sebum (oily secretion) levels and transepidermal water loss (TEWL; constitutive loss of water from the skin surface) will be assessed. Participants will also be asked to fill out a health-related quality of life questionnaire. Safety will be assessed using blood tests, urine analysis, a pregnancy test, and the assessment of vital signs.

**Discussion:**

This trial will utilise high-quality methodologies in accordance with both consolidated standards for reporting trials guidelines and the guidelines for clinical trials of cosmetics products that are aimed at expressions and advertisement approval in Korea. It will evaluate the clinical efficacy and safety of a proposed herbal moisturising cream containing *Arctium lappa L.* seed extract to treat dry skin conditions and provide itch relief. Moreover, we will also employ health-related quality of life questionnaires to assess changes in the quality of life. The results of this study will be used to present the evidence needed to request advertising/display allowances, in compliance with the recently amended Cosmetics Act for advertisement in Korea.

**Trial registration:**

Current Controlled Trials ISRCTN46216631

## Background

Dry skin is usually described as a rough, scaly and flaky surface, which worsens under low humidity conditions and often includes sensations of the loss of elasticity and itch [1]. The causes of dry skin are still not fully understood, and a characterisation and definition of this skin disorder have only recently been completed [2,3]. It is also known as a skin-barrier defect and is mainly attributed to a reduction in sebum secretion and consequent loss of water from the stratum corneum (SC). A healthy SC forms an effective permeability barrier that restricts water loss from the body and blocks the penetration of harmful irritants and allergens [4].

Moisturisers are commonly used by both patients with dry skin conditions and people with healthy skin. However, a lack of knowledge persists regarding the effects of moisturisers on skin barrier function [5]. Early studies conducted on individuals with both healthy and diseased skin showed that some moisturisers tend to weaken the skin barrier function, whereas others may strengthen it, and these discrepant results were assumed to be caused by the varied compositions of moisturisers [5].

Complementary and alternative medicine (CAM) has become increasingly popular among skin disease patients. A 2009 national survey study found that 49.4% of patients with a skin problem in the United States had used a CAM treatment within the previous year, and 6% had used one of these treatments specifically for their skin disease [6]. Several herbal medicines have been promoted for ameliorating various dermatological conditions, and some of them, such as propolis, rosemary, sage, sumac and other extracts, are available in topical formulations and cosmetics [7].

*Arctium lappa L.* (Asteraceae), which is commonly known as burdock, has been popularly used to treat inflammatory disorders and various skin problems and could be a candidate herbal remedy for dry skin conditions [8]. Experimental studies have been conducted to find the active ingredients responsible for increasing the blood circulation to the skin surface, improving the skin quality/texture and curing skin diseases [9]. However, to our knowledge, the clinical trial of a moisturiser containing *Arctium lappa L.* has not yet been conducted.

Recently, regulations on cosmetics were heavily revised to ensure safety in South Korea. Especially in terms of advertising, only cosmetics products that have established evidence using clinical trials are allowed to mark and announce their efficacy. Moreover, according to legislation by the Korean Ministry of Food and Drug Safety (KMFDS), moisturisers cannot be recommended for the treatment of skin diseases [10].

The purpose of this study is to establish the basic clinical efficacy and safety of a proposed herbal moisturiser cream containing *Arctium lappa L.* seed extract in healthy people with dry skin conditions. This trial was designed in accordance with the consolidated standards of reporting trials guidelines (CONSORT) and the guidelines for clinical trials of cosmetics products that are aimed at expression and advertisement approval in Korea [11] and will be conducted as a randomised, double-blind, placebo-controlled trial.

## Methods/Design

**Figure 1:**
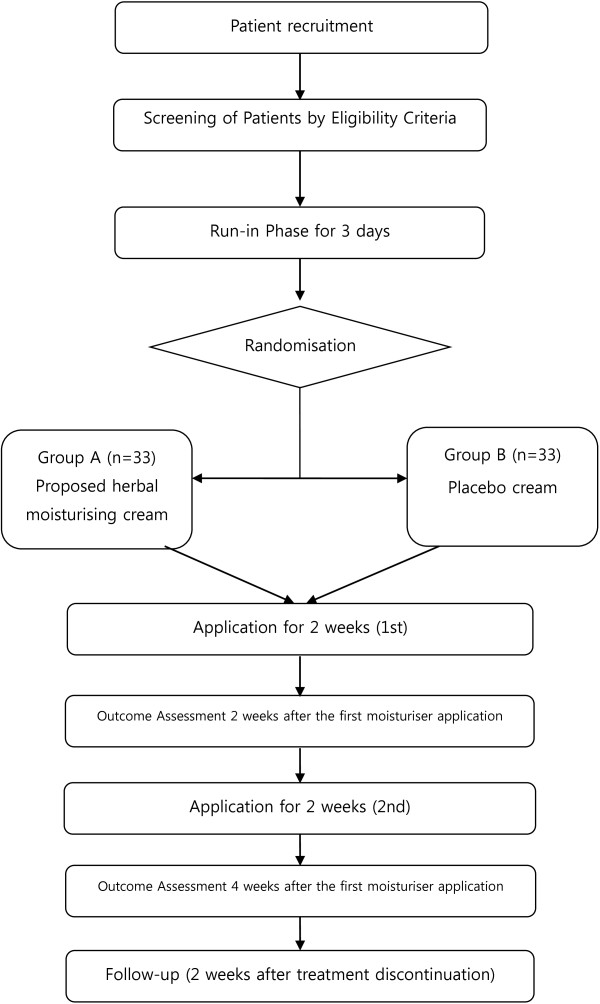
Study flow chart.

A randomised, double-blind, placebo-controlled trial will be conducted at the Wonkwang University Oriental Medical Centre in Gunpo, Korea. Participants fulfilling the eligibility criteria will be selected. Enrolled participants will be randomly allocated to one of two parallel groups: the proposed herbal moisturising cream or the placebo cream. Each participant will be examined for signs and symptoms of skin conditions before and after taking the medication. A follow-up to evaluate the long-term safety will be performed via a phone call (Figure 1).

### Recruitment

We will recruit participants by advertising in local medical centres and on the websites of local medical centres of Gyeonggi province. Respondents will be contacted by clinical trial coordinators (CRC) to determine eligibility via a telephone pre-screen. If an applicant meets the study criteria, he or she will be invited to the clinical research centres to be further examined for eligibility. Korean medicine doctors and internal medicine doctors will conduct the screening together to improve the quality of the eligibility assessment. Korean medicine doctors will manage the screening process. Internal medicine doctors will assess the complete blood cell count (CBC), erythrocyte sedimentation rate (ESR), blood chemistry, urine analysis, and vital signs to exclude patients with serious liver and kidney diseases. Following a pregnancy test, pregnant women will be excluded from the clinical trials.

### Inclusion criteria

Eligible participants of both sexes who are 20 to 65 years of age will be enrolled according to the inclusion and exclusion criteria. We will include participants who present itching or dry skin conditions. The diagnosis of a dry skin condition will be made by the doctor (Korean medicine). Healthy volunteers without skin diseases or any other diseases (acute or chronic) will be enrolled. Participants will be instructed regarding their participation during the run-in, treatment, and follow-up periods. Written informed consent will be obtained from each participant.

### Exclusion criteria

Participants will be excluded if they suffer from any serious medical conditions, such as uncontrolled hypertension, diabetes mellitus requiring insulin injection, past or current malignancy, liver or kidney dysfunction, anaemia, active pulmonary tuberculosis, other severe dermatitis, or any infectious or systemic diseases. The exclusion criteria are as follows: pregnancy, lactation, or planned pregnancy; use of a topical medication containing steroids for the treatment of skin disease more than once per month; participation in the same trial within six months from the interview; hypersensitive skin; skin abnormalities, such as severe acne, erythema, or telangiectasia at the test site; use of the same or similar cosmetics (or pharmaceutical) on the test site within three months of the interview; having undergone a skin peeling or having wrinkles removed within six months of the interview; and any other reasons of unsuitability for the clinical trial at the discretion of the investigator.

Exclusion will be primarily based on information provided by the patients. In addition, additional examinations, such as CBC, ESR, blood chemistry, urine analysis, a pregnancy test, and the assessment of vital signs will be performed before the trial to ensure that the patients do not suffer from any diseases.

We will record the drugs taken by each participant at every visit, and the participants will be asked to notify us of any changes in their medication/supplement regimen. The complete eligibility criteria are provided in Appendix 1.

### Sample size

We wish to estimate the sample size necessary to detect significant differences in skin hydration measured using a Corneometer® 825 (Courage and Khazake, Electronic GmbH, Colongne, Germany) [12], between the experimental and control groups. Sample size was determined based on the results of a recent study [13] in which the mean difference and standard deviation of both groups were 0.94 and 1.22, respectively. The following formula was used to estimate the sample size for a two-group trial:

$$n=\frac{2{\left(Z_{\alpha /2}+Z_{\beta }\right)}^{2}\sigma^{2}}{{\left(\mu_{c}-\mu_{t}\right)}^{2}}$$

Calculations were performed using 80% power, a 5% significance level, and a 20% dropout rate. The required sample size was approximately 27 participants for each group. We plan to enrol 33 participants in each of the two groups to allow for a 20% withdrawal rate, which will ensure that the sample size is in excess of 20, in accordance with the clinical research guidelines of the KMFDS.

### Randomisation and allocation

The randomisation procedure will be guaranteed by Coreana, a consignment organisation. A statistics expert who will have no contact with the study participants will conduct the randomisation of the participants. Participants will be assigned random numbers using a random number generator program (Microsoft Office Excel 2007). These random numbers will be sent to the clinical centre, and the randomisation table will be kept blind by the consignment organisation during the research period. Ultimately, 66 participants will be randomised into two groups. Opaque-sealed envelopes containing serial numbers will be delivered to the clinical centre. An opaque-sealed randomisation table kept by the consignment organisation should be opened as part of the Standard Operating Procedure (SOP). If it has to be opened during an urgent situation involving serious adverse events, the primary investigator must report the incident to the Ethics Committee and the consignment organisation. Before the random assignment, all participants will be informed that they will be assigned to one of two groups. Random allocation will be conducted during the second visit. Random numbers will be matched to their corresponding participants according to the order of the time of second visit. The group assignment results will not be announced to the participants until the last visit of the last participant.

### Blinding

During the trial, investigators will not be in contact with the consignment organisation, the clinical pharmacist, or the statistician. The success of the blinding will be assessed at the last visit of each participant. Researchers who are blinded to the group assignment results will perform the outcome assessment. The blinding procedure will also be verified by the consignment organisation.

### Treatment protocol

Participants will receive the proposed herbal moisturising cream or a placebo cream for four weeks. Application occurs according to the following directions:

Patients in group 1 will receive proposed herbal moisturising cream and instructions on how to apply; they should use an entire packet of the cream (2.00 g) daily. The cream should be applied to the affected areas of skin, to the inner surface of left forearm and to the right side of the face after washing, twice daily (morning and evening), for four weeks.

Patients in group 2 will receive the placebo cream with the same instructions that were provided to group 1.

The defined daily doses (DDDs) of these moisturising creams should be determined based on the specifications and analytical procedures of drug products provided in the KMFDS guidelines. However, no pilot study has previously been reported.

#### Experimental cream

Coreana Cosmetics Co. Ltd. produced the proposed herbal moisturising cream and the placebo cream according to Korea Good Manufacturing Practice (KGMP) standards.

The proposed herbal moisturiser used in this trial is a white, cream-type moisturiser. The proposed herbal moisturising cream is composed of three active ingredients: a controller of sebum biosynthesis; a 70% ethanol extract from the seeds of *Arctium lappa L.*; and vernix caseosa matrix.

#### Placebo cream

Coreana Cosmetics Co. Ltd. developed a homogenous cream that is composed of a base approved by the KMFDS. The colour, form, weight, and scent of the placebo are similar to those of the treatment cream.

### Primary outcome measurement

Skin hydration (Corneometer®), sebum levels (Sebumeter®) and TEWL (Tewameter®) will be assessed as the primary outcome measurements. These parameters will be measured on day 1, day 15 and day 29.

Skin hydration (Corneometer®) will be measured three times at the affected areas of skin, on the inner surface of the left forearm and on right side of the face after washing, and mean values will be adapted for the analysis.

Sebum levels (Sebumeter®) will be measured at the affected areas of skin and on the surfaces of the forehead, cheeks and chin after washing and cleansing (with 70% ethanol). After 60 minutes, the same areas will be measured again, and the differences between two numerical values will be calculated.

After washing, the TEWL (Tewameter®) will be measured three times at the affected areas of skin, on the inner surface of the left forearm and on the right side of the face for 30–50 seconds, and the mean values will be adapted for the analysis.

### Secondary outcomes

Secondary outcome measurements include the SC moisture levels (Corneofix®), visual analogue scale (VAS)-xerosis, and VAS-itching. These parameters will be measured on days 1, 15 and 29. The EuroQoL 5-Dimension (EQ-5D), the Health Utilities Index Mark 3 (HUI3), and the Dermatology Life Quality Index (DLQI) will be administered on day 1 and day 29 as measures of the health outcomes. The data collection schedule is detailed in Table 1.

**Table 1:** Data collection schedule

**Period**	**Screening**	**Treatment period**			**Follow-up**
**Visit**	**1st**	**2nd**	**3rd**	**4th**	
	**(D −3)**	**(D 1)**	**(D 15)**	**(D 29)**	**(D 43)**
Informed consent	○				
Demographic characteristics	○				
Medical History	○				
Safety assessment	○	○	○	○	
Skin hydration		○	○	○	
Sebum level		○	○	○	
Transepidermal water loss		○	○	○	
VAS (xerosis, itching)		○	○	○	
Dermatology life quality index		○		○	
EQ-5D		○		○	
Health utilities index mark 3		○		○	
Adverse effects			○	○	○
Blinding assessment				○	

### Patient safety

All patients will undergo routine testing that includes the following assays: CBC, ESR, blood chemistry, pregnancy test, and urine analysis before randomisation and immediately after completing the treatment. Vital signs will be measured at every visit. These tests help identify and exclude patients with serious liver and kidney disease or other severe illness. Investigators will assess a significant result in these tests according to explicit criteria defining the incidence and intensity of adverse events. In addition, pictures of the skin lesions will be recorded as a means of evaluating the adverse events.

### Statistical analysis

Analyses will be performed for two populations: 1) an intention-to-treat population consisting of all randomised participants who have at least one measurable outcome report following treatment (missing data are replaced with the last observed values) and 2) a per-protocol population including only participants without any major protocol deviations. All baseline characteristics of both groups will be described. All of the main analyses will be based on the intention-to-treat population and conducted using the LOCF (last observation carried forward) imputation method. For primary outcome measures, the mean difference from baseline values at the end of treatment will be compared using independent t-tests (expected noncentrality parameter = 2.90, critical t = 2.00, power = 0.80). Paired t-tests or non-centrality Wilcoxon rank sum tests will be used to compare the safety variables. Repeated measures ANOVA (analysis of variance) or repeated measures ANCOVA (analysis of covariance) tests will also be performed as within-group and between-group analyses. Baseline data will be collected on each participant during the randomisation and can be used to describe the population of patients, to compare the treatment groups, to achieve balance in the randomisation, to adjust for possible confounding of the prognostic factors, and to undertake subgroup analyses. We will exercise caution when drawing conclusions from the subgroup findings.

Statistical analyses will be performed using the STATA statistical package program (ver. 11.2), and the level of significance will be established at α = 0.05.

### Data and safety monitoring

To maintain the quality of this trial, monitoring will be conducted by Coreana, a consignment organisation that is located in Chungnam, Korea. Investigators can also convene to discuss practical issues that they might encounter, such as dealing with serious adverse events, revising the protocol, and addressing certain important issues that might be raised by investigators and participants. We define adverse events as unintended signs, symptoms, or diseases occurring after treatment that may or may not be related to the intervention. The safety assessment is based primarily on the frequency of adverse events, including all serious adverse events. Information on the adverse events will be summarised by presenting the number and percentage of participants experiencing any adverse events. During the trial, all adverse events will be observed in detail and documented using case report forms (CRFs).

### Ethics

Written consent will be obtained from each participant. This study protocol was approved by the institutional review board (IRB) of the Wonkwang University Oriental Medical Centre.

## Discussion

Dry skin is a common symptom of a number of skin diseases, including atopic dermatitis/eczema, ichthyosis, irritant contact dermatitis, psoriasis and asteatotic eczema, and can also be seen in healthy individuals [1,14]. It is more prevalent in geriatric populations than in younger patients and could be affected by environmental factors, such as frequent washing, the use of harsh detergents and exposure to low-humidity air [4]. These factors can influence moisturiser studies, and so in this study, certain clinical and habitual factors, such as the menopausal state, bathing habits and the treatment experiences of other skin diseases will be included in the baseline assessments.

Prior studies of moisturisers have mainly focused on short-term results using measurement devices, as it is possible to attain an improvement in the skin moisture shortly after a single application. Nevertheless, long-term studies are important to assess the maintenance and enhancement of this effect [15], and we designed a 6 week study period for observing the long-term results.

Although dry skin is not a life-threatening condition, the symptom is apparently unpleasant and can lead to deterioration in the quality of life [14], which should also be considered during moisturiser studies. Therefore, in addition to the primary outcome and secondary outcomes that were measured using instruments, the EQ-5D, HUI-III, DLQI self-reported measures were also included to examine changes in the general and disease-specific quality of life.

Herbal products have gained increasing popularity and are now used by approximately 20% of the population in the United States [16]. Similar usage patterns can be observed for cosmetics, and due to the harmful effects of chemicals, studies and markets have focused on herbal cosmetics [17]. The South Korean cosmetics report revealed that sales of herbal cosmetics had expanded more than 2 fold over the last 5 years, accounting for 23.5% of the total cosmetics market in 2010 [18]. In this context, various herbal medicines, such as A. lappa, have been studied as potential cosmetics products. A recent experimental study reported that an A. lappa fruit extract-containing formulation improved the metabolism of the dermal extracellular matrix and lead to a visible reduction in wrinkles [19]. However, side effects have also been reported for A. lappa, such as contact dermatitis [20] and anaphylaxis [21]. Therefore, the potential for rare but dangerous side effects indicate that safety issues should also be examined during clinical trials. In this study, we will perform blood tests, urine analysis and adverse event case reports to confirm the safety of the proposed herbal moisturiser.

In conclusion, the present trial includes two unique features. First, we developed a protocol in conjunction with the recommendations of the consolidated standards of reporting trials (CONSORT) checklists for herbal interventions and the clinical trial guidelines of the KMFDS for cosmetics products. Second, we conducted a systematic review before developing the study design and adopted outcome measurements following the results [22].

This study will demonstrate the efficacy and safety of the proposed herbal moisturiser in healthy people with dry skin conditions.

## Appendix 1: Eligibility Criteria

### Inclusion criteria

1. Volunteer individuals aged 20 to 65 years, either sex.

2. Written and informed consent.

3. Healthy volunteers without skin disease or any other diseases (acute or chronic).

4. Diagnosed with dry skin by the doctor (Korean medicine) or presenting with itching or a dry skin condition.

5. Available for follow-up observations during the clinical trial period.

### Exclusion criteria

1. Pregnancy, lactation, or planned pregnancy.

2. The use of a topical medication containing steroids to treat skin disease more than once a month.

3. Participation in the same trial within six months of the interview.

4. Hypersensitive skin.

5. Skin abnormalities, such as severe acne, erythema, or telangiectasia at the test site.

6. Use of the same or similar cosmetics (or pharmaceuticals) at the test site within three months of the interview.

7. Having undergone a skin peeling or wrinkle removal procedure during the six months prior to the interview.

8. Any other reasons of unsuitability for the clinical trial at the discretion of the investigator.

## Abbreviations

ANCOVA: Analysis of covariance; ANOVA: Analysis of variance; CAM: Complementary and alternative medicine; CBC: Complete blood cell count; CONSORT: Consolidated Standards of Reporting Trials; CRC: Clinical research coordinators; CRF: Case report form; DDDs: Defined daily doses; DLQI: Dermatology life quality index; ESR: Erythrocyte sedimentation rate; EQ-5D: EuroQol 5-dimension; HUI3: Health utilities Index mark 3; IRB: Institutional review board; KGMP: Korea good manufacturing practice; KMFDS: Korean ministry of food and drug safety; LOCF: Last observation carried forward; SOP: Standard operating procedure; TEWL: TransEpidermal water loss; SC: Stratum corneum; VAS: Visual analogue scale.

## Competing interests

The authors disclose the following: GTL, YKY, and KKL are employees of Coreana Songpa Research and Development Center in Coreana Cosmetics Co. Ltd. The company has been participating in Korean Healthcare Technology R&D Project mentioned in acknowledgements as a collaborative partner for developing cosmetic ingredients and designs. The remaining authors declare that they have no competing interests.

## Authors’ contributions

All authors participated in the conception and design of the trial. NKK is the principal investigator of this study. He drafted the protocol. DHL, GWJ, and NKK wrote the final manuscript. ESS, JTH, GTL, YKY, and KKL contributed to the research design and made critical revisions. All authors read and approved the final manuscript.

## Pre-publication history

The pre-publication history for this paper can be accessed here:

http://www.biomedcentral.com/1472-6882/13/330/prepub
